# Influence of nicotine on choline-deficient, L-amino acid-defined diet-induced non-alcoholic steatohepatitis in rats

**DOI:** 10.1371/journal.pone.0180475

**Published:** 2017-06-29

**Authors:** Hiroyuki Kanamori, Yukiomi Nakade, Taeko Yamauchi, Kazumasa Sakamoto, Tadahisa Inoue, Takaya Yamamoto, Yuji Kobayashi, Norimitsu Ishii, Tomohiko Ohashi, Kiyoaki Ito, Yoshio Sumida, Haruhisa Nakao, Yoshitaka Fukuzawa, Masashi Yoneda

**Affiliations:** Division of Gastroenterology and Hepatology, Department of Internal Medicine, Aichi Medical University, Nagakute, Aichi, Japan; Medizinische Fakultat der RWTH Aachen, GERMANY

## Abstract

Nicotine, a major compound in cigarette smoke, decreases food intake and body weight gain in mammals; however, the influence of nicotine on the progression of non-alcoholic steatohepatitis (NASH) remains controversial. This study aimed to investigate the effect of nicotine on NASH in rat models. Male Wistar rats were fed choline-deficient, l-amino acid-defined (CDAA) diet and treated with nicotine or saline. Food intake, body weight gain, presence of hepatic steatosis, inflammation, and fibrosis were assessed 6 weeks after the rats were fed CDAA diet. Hepatic branch vagotomy was performed to elucidate the mechanism through which nicotine affected steatohepatitis.

CDAA diet induced hepatic steatosis, inflammation, and fibrosis, as well as increased the expression of inflammation-related genes. Conversely, nicotine significantly attenuated food intake, body weight gain, and inhibited the CDAA-diet-induced hepatic steatosis, inflammation, and fibrosis, together with increased expression of inflammation-related genes. Hepatic branch vagotomy by itself decreased food intake, body weight gain, and attenuated the CDAA-diet-induced hepatic steatosis, but not inflammation. However, nicotine did not change the food intake, body weight gain, and CDAA diet-induced hepatic steatosis and inflammation in vagotomized rats. These results suggest that nicotine attenuates the CDAA-diet-induced hepatic steatosis and inflammation through the hepatic branch of the vagus nerve in rats.

## Introduction

With the increased occurrence of the metabolic syndrome, the incidence of non-alcoholic fatty liver disease (NAFLD) has also dramatically increased in recent years, becoming a major health issue and the most common liver disease worldwide [1, 2]. Non-alcoholic steatohepatitis (NASH), which is characterized by steatosis, necroinflammation, and cytopathic changes, causes liver cirrhosis and lies within the NAFLD spectrum [3]. Although the pathogenesis of NASH remains elusive, an accumulation of excess lipids in the liver may be a prerequisite for developing NASH [4].

Several therapeutic approaches for treating NASH have been reported; however, the only promising therapy thus far is reduction of body weight [5]. Nicotine, a major component of cigarettes, exerts various physiological effects in mammals. Nicotine administration in rodents decreases food intake and body weight gain, and increases energy expenditure; conversely, withdrawal of nicotine causes hyperphagia and body weight gain [6, 7]. Long-term administration of nicotine can increase insulin sensitivity in the obese rats with diabetes by decreasing glycogen content in the liver [8]. Conversely, nicotine can decrease hepatic blood flow in rats via the activity of endogenous endothelin-1 [9]. Carbon tetrachloride-induced acute liver injury in rats is exacerbated by the administration of nicotine [10]. Thus, nicotine exerts positive as well as negative effects on the physiology and pathophysiology; however, it is controversial whether nicotine has a negative impact on NASH.

A previous study on rats demonstrated that chronic administration of nicotine decreases food intake, obesity, and hepatic steatosis [11]. Furthermore, nicotine improves the serum lipid profile, and decreases inflammation and endoplasmic reticulum stress in rats with diet-induced obesity [11]. On the contrary, the hepatic branch of the vagus nerve plays an important role in regulating food intake [12].The hepatic vagal nerve is one of the sensors of nicotine in the hepatoportal region [13]. In this study, we examined the influence of nicotine on food intake, body weight gain, histological changes in the liver, lipid metabolism, and inflammation in rats with choline-deficient, l-amino acid-defined (CDAA) diet-induced NASH. We also examined the involvement of the hepatic vagal nerve in nicotine-induced changes in food intake, hepatic steatosis, and inflammation in CDAA diet-fed rats.

## Materials and methods

### Substances and treatments

Nicotine was purchased from Sigma Aldrich (St. Louis, MO). The CDAA diet was used to induce NASH in the rat model; the choline-sufficient, l-amino acid-defined (CSAA) diet was used as a control diet. Both the diets were purchased in a powdered form from the Oriental Yeast Co. (Tokyo, Japan). An antibody against CD68, which is a marker for macrophages [14], was purchased from AbD Serotec (Oxford, UK). Anti-α-smooth muscle actin (α-SMA) monoclonal antibody was purchased from Abcam (clone 1A4, Cambridge, MA).

### Animal model and experimental design

Six-week-old male Wistar rats were purchased from Japan SLC Inc. (Hamamatsu, Japan). The rats were acclimatized in cages under conditions of controlled temperature (22–24°C), humidity, and illumination (12 hour light cycle starting at 6:00 am) for at least 7 days before undergoing the experiments. After 1-week acclimatization period on a basal diet (Oriental Yeast), the rats were divided into four groups (6–8 rats/group) and then osmotic minipumps (Alzet model 2006, Alza; Palo Alto, CA), containing nicotine or saline, were implanted by an interscapular incision into the subcutaneous space of the back under isoflurane anesthesia. Next, the rats were fed with CDAA or CSAA diet, and nicotine (12 mg/kg/day) or saline (3.6 μL/day) was co-administered continuously via the minipumps for 6 weeks. The dose of nicotine was determined according to a previous report [15]. All the rats were provided with free access to water and experimental diets. Body weights and food intake in each group of rats were recorded every week and every day, respectively. Protocols describing the use of rats were approved by the Institutional Animal Care and Use Committee of the Aichi Medical University and were in accordance with the National Institutes of Health "Guide for the Care and Use of Laboratory Animals." All rats were euthanized 6 weeks after the commencement of CSAA or CDAA diet using CO_2_ inhalation. The liver from all the rats was immediately collected, weighted, and stored at −80°C for RNA extraction. Blood samples were collected from the left ventricle and centrifuged; plasma was stored at −80°C for subsequent analyses.

To investigate nicotine-induced alteration of CDAA-induced steatohepatitis, hepatic branch vagotomy or a sham operation was performed under isoflurane anesthesia 1 week before the implantation of osmotic minipumps. Hepatic branch vagotomy was performed under a dissection microscope by selective sectioning of the hepatic branch of the vagal nerve, which diverges from the anterior vagal trunk a few millimeters proximal to the cardia. One week after hepatic branch vagotomy, rats were subcutaneously implanted in the back with osmotic minipumps containing nicotine or saline; the procedure was conducted under isoflurane anesthesia. Next, rats were administered the CDAA diet for 6 weeks, after which the effects of hepatic branch vagotomy on body weight gain, hepatic steatosis, and inflammation were assessed.

### Assessment of hepatic steatosis, inflammation, fibrosis, and Kupffer cells

Hepatic steatosis was assessed by monitoring the levels of hepatic triglycerides (TG), free fatty acid (FFA), and Oil Red O staining. Stored liver samples (100 mg) were lysed and homogenized, using a Polytron homogenizer (NS-310E; MicroTech Nichion, Tokyo, Japan), for 1 min in 2 mL solution containing 150 mM NaCl, 0.1% TritonX-100, and 10 nM Tris. TG and FFA levels in the liver were measured using the Triglyceride Detection Kit (Wako, Osaka, Japan) and non-esterified fatty acid Detection Kit (Wako, Osaka, Japan), respectively. Oil Red O staining was also performed on the frozen liver tissue samples using standard techniques. To evaluate the extent of hepatic inflammation and fibrosis, hematoxylin and eosin staining and Masson’s trichrome staining was performed on formalin-embedded 5-μm thick sections of the liver tissue respectively. Levels of TG and alanine aminotransferase (ALT) in the serum were measured using commercially available kits (Wako). The degree of hepatic steatosis, inflammation, and hepatocellular ballooning were assigned a score from 0 to 3, based on a histological scoring system for NAFLD established by Kleiner et al. [16]. For semiquantitative morphometric analysis of hepatic steatosis and fibrosis, the area stained with Oil Red O and Masson’s trichrome stains was quantified by an computerized image analysis system, Image-Pro Plus version 4.5 (Media Cybernetics, Silver Spring, MD), at 50× magnification using five randomly selected fields per section, respectively. Formalin-fixed paraffin-embedded liver sections were used for immunohistochemical analysis. After deparaffinization and rehydration in xylene and a graded series of alcohol, endogenous peroxidase was blocked with hydrogen peroxide. Non-specific binding was blocked with 10% normal goat serum in phosphate buffered saline. After blocking, the sections were incubated overnight with the anti-CD68 mouse monoclonal antibody diluted at 1:200, and the anti-α-SMA monoclonal antibody diluted at 1:1000. CD68-positive and αSMA-positive cells were counted under the light microscope using five fields per slide at 50× magnification and average counts were noted.

### Analysis of mRNA from the liver tissues by real-time polymerase chain reaction

The frozen liver specimens were pulverized in TRIzol (Life Technologies, Tokyo, Japan). RNA extraction was performed using the RNeasy Mini Kit (Qiagen, Tokyo, Japan). RNA was resuspended in 40 μL RNase-free water and quantified using the OD260 spectrophotometer and low-mass gel electrophoresis (Invitrogen, Tokyo, Japan). Total RNA was reverse-transcribed to cDNA using the High Capacity cDNA Reverse Transcription Kit (Applied Biosystems, Foster City, CA) according to the manufacturer’s instructions. Real-time polymerase chain reaction (qPCR) was conducted on Step One Sequence Detection System (Applied Biosystems) using TaqMan Gene Expression Assays; the TaqMan probe IDs are listed in Table 1. TaqMan Universal PCR Master Mix (Applied Biosystems) was used according to the manufacturer’s instructions. The expression of the genes involved in the hepatic lipid metabolism, such as apolipoprotein B (*apoB*), microsomal triglyceride transfer protein (*MTTP*), stearoyl-element binding protein 1 (*SREBP1*), acyl-coenzyme A oxidase 1 (*ACOX1*), and elongation of very long chain fatty acids-like 6 (*Elovl6*), was assessed. To examine the degree of the inflammatory response, we also assessed the expression of the macrophage surface marker, *CD68*, and that of tumor necrosis factor-α (*TNF-*α), interleukin (*IL*)*1β*, and *IL6* at the mRNA level. To examine the involvement of apoptosis, we also assessed the expression of Bcl2-associated X protein (*Bax*) and caspase 3 (*Cas3*) at the mRNA levels. To examine the hepatic stellate cell activation, alpha-smooth muscle actin 2 (*ACTA2*) expression was measured at the mRNA level. The relative levels of mRNA transcripts were quantified using the 2^-ΔΔCT^ method. The variation in the amount of available DNA in the samples was controlled by normalizing the levels of the target sequence to those of the endogenous control, 18s ribosomal RNA, using the 18s rRNA TaqMan Control Reagent Kit (Applied Biosystems). The expression of target genes was normalized to that of 18s ribosomal RNA in each sample, and expressed as the magnitude of change relative to gene expression after 6 weeks of feeding CSAA or CDAA diet co-administered with saline. The conditions for qPCR were similar to those outlined in a previous report [17].

**Table 1:** Taqman probe ID for real time PCR.

Gene	Gene bank number	Taqman ID
*ApoB*	NM_019187	Rn01499054_m1
*MTTP*	NM_001107727	Rn01522963_m1
*SREBP1*	NM_001276708	Rn01495769_ m1
*Elovl6*	NM_134383	Rn00592815_m1
*ACOX1*	NM_017340	Rn01759925_g1
*CD68*	NM_001031638	Rn01495634_g1
*TNF-α*	NM_012675	Rn00562055_m1
*IL1β*	NM_031512	Rn00580432_m1
*IL6*	NM_012589	Rn01410330_m1
*Bax*	NM_017059	Rn01480161_g1
*Cas3*	NM_012922	Rn00563902_m1
*Acta2*	NM_031004.2	Rn01759928_g1

### Data analysis

All data are expressed as means ± SE. The values in the two groups were compared using Student’s *t*-test. Multiple group comparison was performed using analysis of variance (ANOVA) followed by Fisher’s protected least significant difference post-hoc test; a p value < 0.05 was considered statistically significant.

## Results

**Fig 1:**
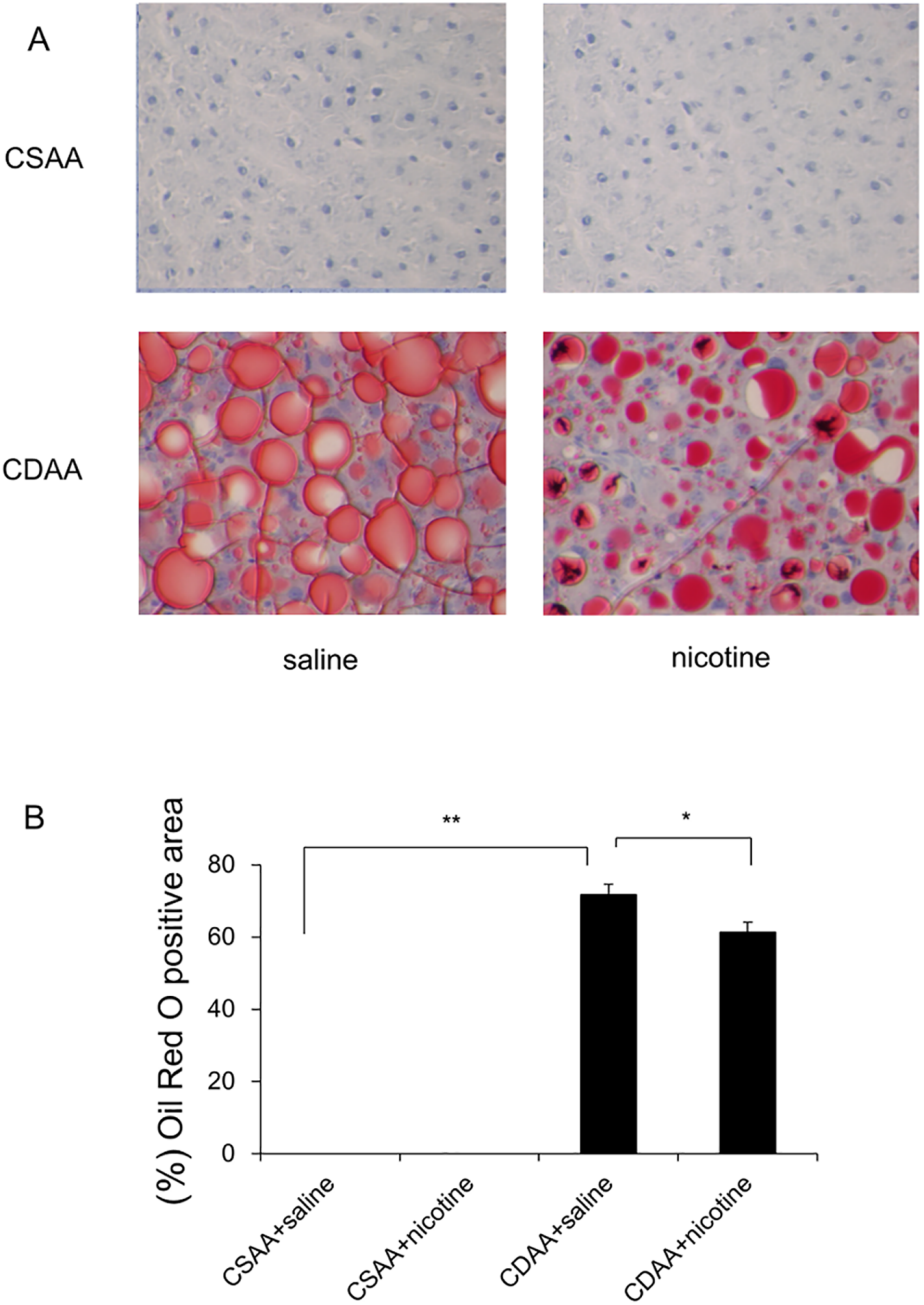
Representative photomicrographs showing the effects of CSAA and CDAA diet on liver histology in rats. Frozen liver tissues were stained with Oil Red O (A). Rats were fed CSAA diet and CDAA diet, co-administered with saline, or CSAA diet and CDAA diet, co-administered with nicotine, for 6 weeks. Original magnification, ×200. Quantitative analysis of changes in Oil Red O positive areas in respective groups (B). Data are expressed as means ± SE of six to eight rats. (* p < 0.05, ** p < 0.01 compared with the respective group).

The CDAA-diet-fed rats co-administered with saline showed a significantly reduced body weight gain compared to that of CSAA-diet-fed rats co-administered with saline (Table 2), whereas the food intake of the CDAA diet-fed rats co-administered with saline was comparable to that of CSAA-diet-fed rats co-administered with saline (Table 2). While the CSAA diet did not induce hepatic steatosis, the CDAA diet induced hepatic steatosis, which was assessed by the levels of hepatic TG and Oil Red O staining (Table 2, Table 3, Fig 1A and 1B). The levels of hepatic TG and FFA in CDAA-diet-fed rats co-administered with saline were significantly higher than those in CSAA-diet-fed rats co-administered with saline (Table 2). On the contrary, the serum levels of TG in CDAA-diet-fed rats co-administered with saline were significantly lower than those in CSAA-diet-fed rats co-administered with saline (Table 2). Serum levels of ALT in CDAA-diet-fed rats co-administered with saline were significantly higher than those in CSAA-diet-fed rats co-administered with saline (Table 2).

**Table 2:** Clinical characteristics of rats after 6 weeks feeding of experimental diets.

Group	n	BodyWeight gain(g)	Food Intakes (g/day)	Serum TG(mg/dl)	Hepatic TG(mg/dl)	Hepatic FFA (mEq/l)	Serum ALT (IU/L)
CSAA + saline	6	145 ± 5.0	11.9 ± 1.9	150 ± 14	67 ± 5.8	0.7 ± 0.1	24± 2.0
CSAA + nicotine	6	136 ± 2.7^a^	10.5 ± 0.5^a^	174 ± 29	68 ± 4.8	0.8 ± 0.1	30 ± 4.2
CDAA + saline	7	82 ± 3.2^b^	12.3 ± 1.5	25 ± 1.2^a^	653 ± 129^a^	1.3 ± 0.3^a^	204 ± 14.7^a^
CDAA + nicotine	8	68 ± 3.8^c^	10.1 ± 1.7 ^c^	24 ± 1.5^a^	440 ± 20^c^	0.3 ± 0.1^c^	190 ± 15.7 ^a^

Data are expressed as means ± SEM; Statistical comparison were made using one-way ANOVA

^a^; significant different from CSAA+saline 6 weeks (p < 0.05)

^b^; significant different from CSAA+saline 6 weeks (p < 0.01)

^c^; significant different from CDAA+saline 6 weeks (p < 0.05)

**Table 3:** NAFLD activity scores (NAS) of rats fed experimental diets with nicotine.

Group	n	steatosis	inflammation	Hepatocyte ballooning
CSAA + saline	6	0 ± 0	0 ± 0	0 ± 0
CSAA + nicotine	6	0 ± 0	0 ± 0	0 ± 0
CDAA + saline	7	2.667 ± 0.042^a^	1.600 ± 0.225^a^	0.233 ± 0.033^a^
CDAA + nicotine	8	2.375 ± 0.139^b^	1.200 ± 0.256 ^b^	0.100 ± 0.038^b^

Data are expressed as means ± SEM; Statistical comparison were made using one-way ANOVA

^a^; significantly different from CSAA+saline (p < 0.01)

^b^; significantly different from CDAA+saline (p < 0.05)

Nicotine administration attenuated food intake and body weight gain; however, it could not change the levels of serum ALT and hepatic TG in CSAA-diet-fed rats (Table 2). Nicotine administration significantly attenuated food intake, body weight gain, and hepatic fat deposition, which was confirmed by the area stained with Oil Red O, and increased levels of hepatic TG and FFA in CDAA-diet-fed rats (Table 2, Fig 1A and 1B). The decreased serum levels of TG and increased serum levels of ALT were not significantly altered by nicotine administration in CDAA-diet-fed rats (Table 2).

**Fig 2:**
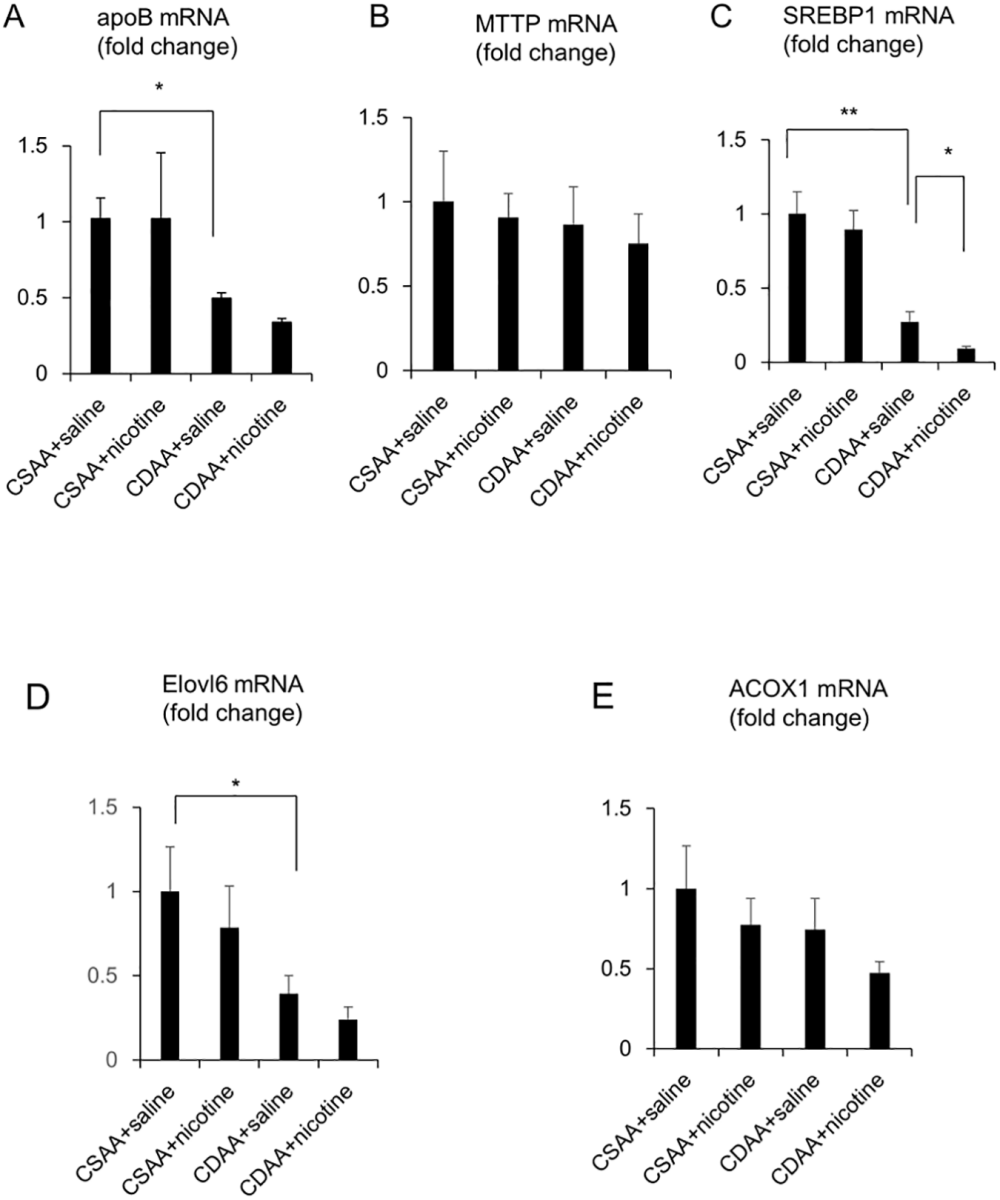
Evaluation of hepatic lipid metabolism-related gene expressions in CSAA- and CDAA- diet-fed rats. The relative mRNA expression of *apoB* (A), *MTTP* (B), *SREBP1* (C), *Elovl6* (D) and *ACOX1* (E) were evaluated 6 weeks after the commencement of CSAA and CDAA diets. The expression of target genes was normalized to that of 18s ribosomal RNA in each sample, and expressed as the magnitude of change relative to gene expression after 6 weeks of feeding CSAA diet co-administered with saline. The values are means ± SE of six to eight rats. Statistically significant differences between the respective groups are indicated as * p < 0.05, ** p < 0.01.

CDAA diet significantly attenuated the hepatic expression of *apoB* at the mRNA levels, which was not changed by nicotine administration (Fig 2A). CDAA diet could not change the hepatic expression of *MTTP* at the mRNA levels, which was also not changed by nicotine administration (Fig 2B). While nicotine administration could not change the hepatic expression of *SREBP1* at the mRNA levels in control diet-fed group, CDAA diet significantly decreased the hepatic expression of *SREBP1* at the mRNA levels, which was significantly attenuated by nicotine administration, in the CDAA diet-fed group (Fig 2C). While nicotine administration could not change the hepatic expression of *Elovl6* at the mRNA levels in the control diet, CDAA diet significantly decreased the hepatic expression of *Elovl6* at the mRNA levels, which tended to be decreased by nicotine administration, in the CDAA diet-fed group (Fig 2D). CDAA diet could not change the hepatic expression of *ACOX1* at the mRNA levels, which was also not changed by nicotine administration (Fig 2E).

**Fig 3:**
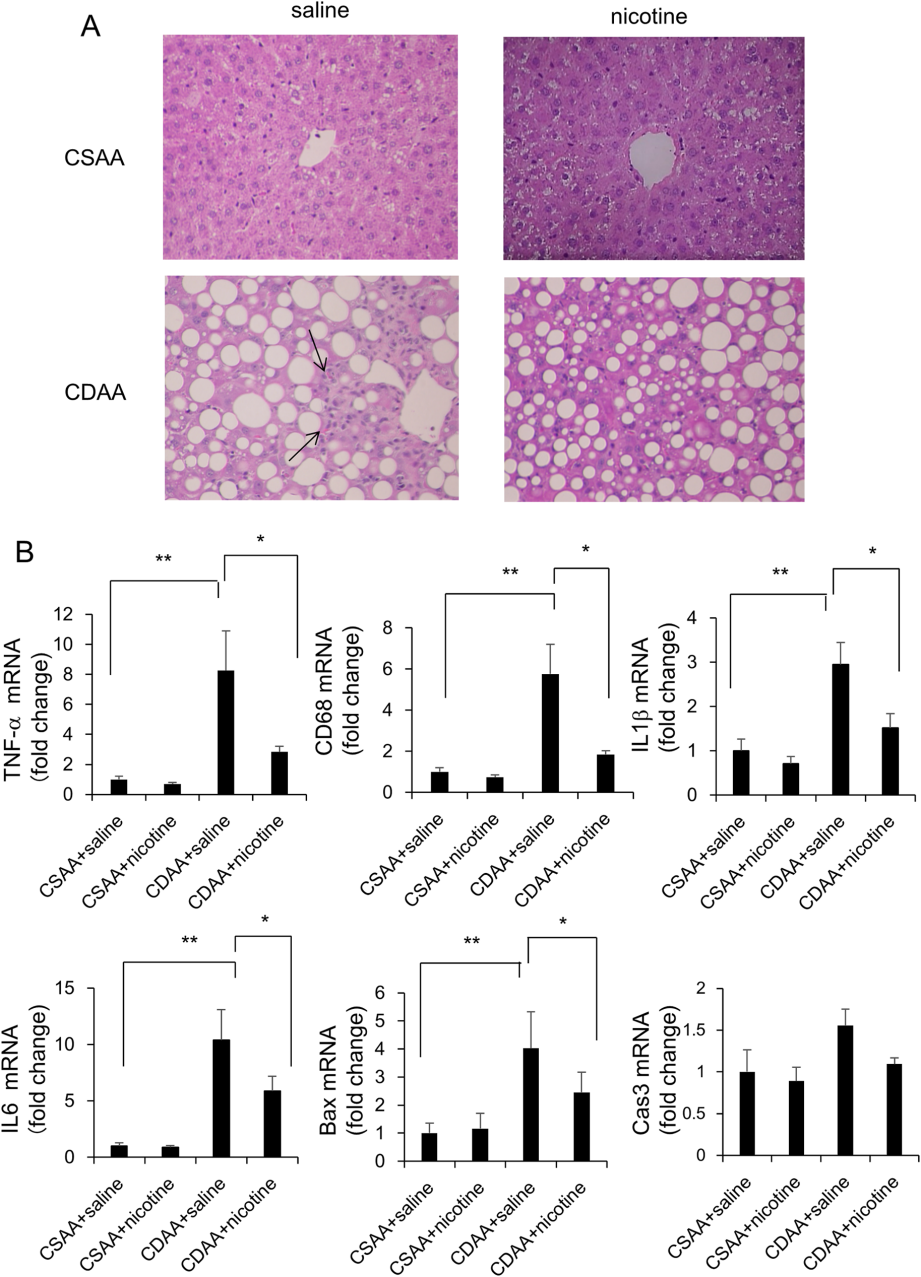
Evaluation of liver inflammation-related parameters in CSAA- and CDAA-diet-fed rats. Representative photomicrographs showing the inflammation of CSAA and CDAA diet on liver histology in rats. Paraffin-embedded liver sections were stained with H-E staining. Rats were fed CSAA diet and CDAA diet, co-administered with saline, or CSAA diet and CDAA diet, co-administered with nicotine, for 6 weeks. Arrows indicate the inflammatory foci in the liver. Original magnification, ×200 (A). The relative expression of *TNF-α*, *CD68*, *IL1β*, *IL6*, *Bax* and *Cas3* mRNA in the liver was evaluated 6 weeks after feeding (B). The expression of target genes was normalized to that of 18s ribosomal RNA in each sample, and expressed as the magnitude of change relative to gene expression after 6 weeks of feeding CSAA diet co-administered with saline. Data are expressed as means ± SE of six to eight rats. (* p < 0.05, ** p < 0.01 compared with the respective groups).

**Fig 4:**
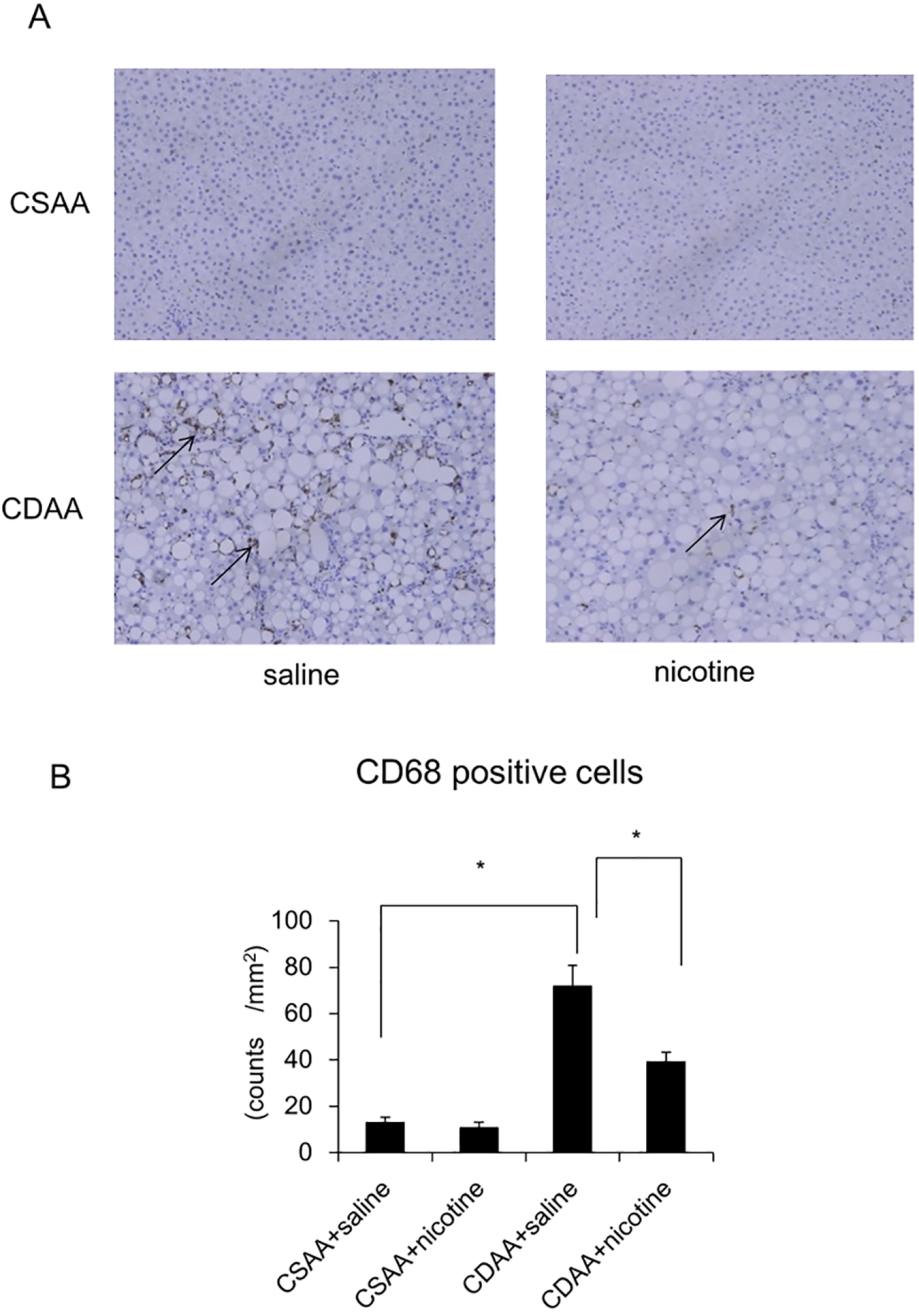
Evaluation of immunohistochemical staining of hepatic CD68-positive cells in CSAA- and CDAA-diet-fed rats. Representative photomicrographs showing the hepatic CD68-positive cells in CSAA- and CDAA-diet-fed rats co-administered the saline control or nicotine for 6 weeks. Arrows indicate CD68-positive cells. (A).Quantitative analysis of changes in the numbers of CD68-positive cells in the respective groups (B). Original magnification, ×100. Data are expressed as means ± SE of six to eight rats. (* p < 0.01 compared with the respective groups).

**Fig 5:**
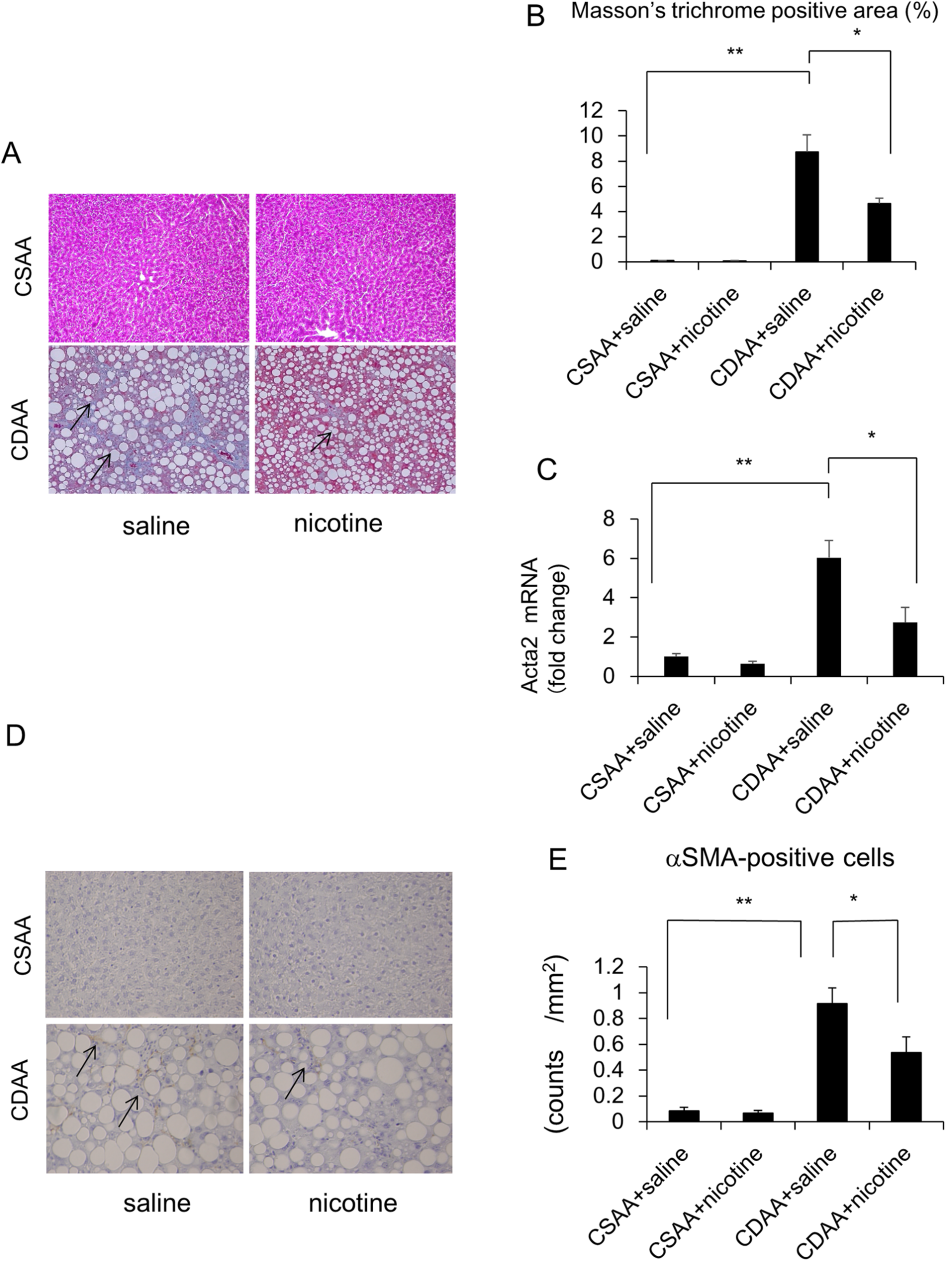
Evaluation of liver fibrosis-related parameters in CSAA- and CDAA-diet-fed rats. Representative photomicrographs showing the fibrosis of CSAA and CDAA diet on liver histology in rats. Liver tissues were stained with Masson’s trichrome staining. Rats were fed CSAA diet and CDAA diet, co-administered with saline, or CSAA diet and CDAA diet, co-administered with nicotine, for 6 weeks. Arrows indicate the hepatic fibrosis in the liver. Original magnification, ×100 (A). Quantitative analysis of changes in the Masson’s trichrome staining blue colored area in the respective groups (B). The expression of *Acta2* was normalized to that of 18s ribosomal RNA in each sample, and expressed as the magnitude of change relative to gene expression after 6 weeks of feeding CSAA diet co-administered with saline (C). Evaluation of immunohistochemical staining of hepatic αSMA-positive cells in CSAA- and CDAA-diet-fed rats co-administered the saline control or nicotine for 6 weeks. Arrows indicate αSMA-positive cells (D). Quantitative analysis of changes in the numbers of αSMA-positive cells in the respective groups (E). Data are expressed as means ± SE of six to eight rats. (* p < 0.05, ** p < 0.01 compared with the respective groups).

While the CSAA diet could not induce hepatic inflammation, CDAA diet induced hepatic inflammation, as assessed by the inflammation score (Table 3, Fig 3A). CDAA diet increased the hepatic expression of *TNF-α*, *CD68*, *IL1β*, *IL6*, *Bax*, and *Cas3* at the mRNA level, as well as the number of CD68-positive cells, compared to that in the CSAA diet-fed rats co-administered with saline (Fig 3B, Fig 4A and 4B). While ballooned hepatocytes were not observed in CSAA diet-fed rats treated with saline, CDAA diet-fed rats showed presence of the ballooned hepatocytes (Table 3). Nicotine administration did not affect hepatic inflammation, hepatocyte ballooning scores, expression of *TNF-α*, *CD68*, *IL1β*, *IL6*, *Bax*, and *Cas3* at the mRNA level, or number of CD68-positive cells in CSAA-diet-fed rats (Table 3, Fig 3A and 3B, Fig 4A and 4B), whereas it significantly attenuated these parameters in CDAA-diet-fed rats. (Table 3, Fig 3A and 3B, Fig 4A and 4B). Nicotine administration also tended to decrease *Cas3* expression at the mRNA level (Fig 3B). While the CSAA diet did not induce hepatic fibrosis, CDAA diet induced hepatic fibrosis as assessed by Masson’s trichrome stain-positive area (Fig 5A and 5B). CDAA diet increased the hepatic expression of *Acta2* at the mRNA level, as well as the number of α-SMA-positive cells, as compared to those in the CSAA-diet-fed rats co-administered with saline (Fig 5C–5E). Nicotine administration did not affect hepatic fibrosis, *Acta2* expression at the mRNA level, or number of α-SMA-positive cells in CSAA-diet-fed rats (Table 3, Fig 5A–5E). However, nicotine administration significantly attenuated hepatic fibrosis and *Acta2* expression at the mRNA level, but not the number of α-SMA-positive cells in CDAA-diet-fed rats (Fig 5A–5E).

**Fig 6:**
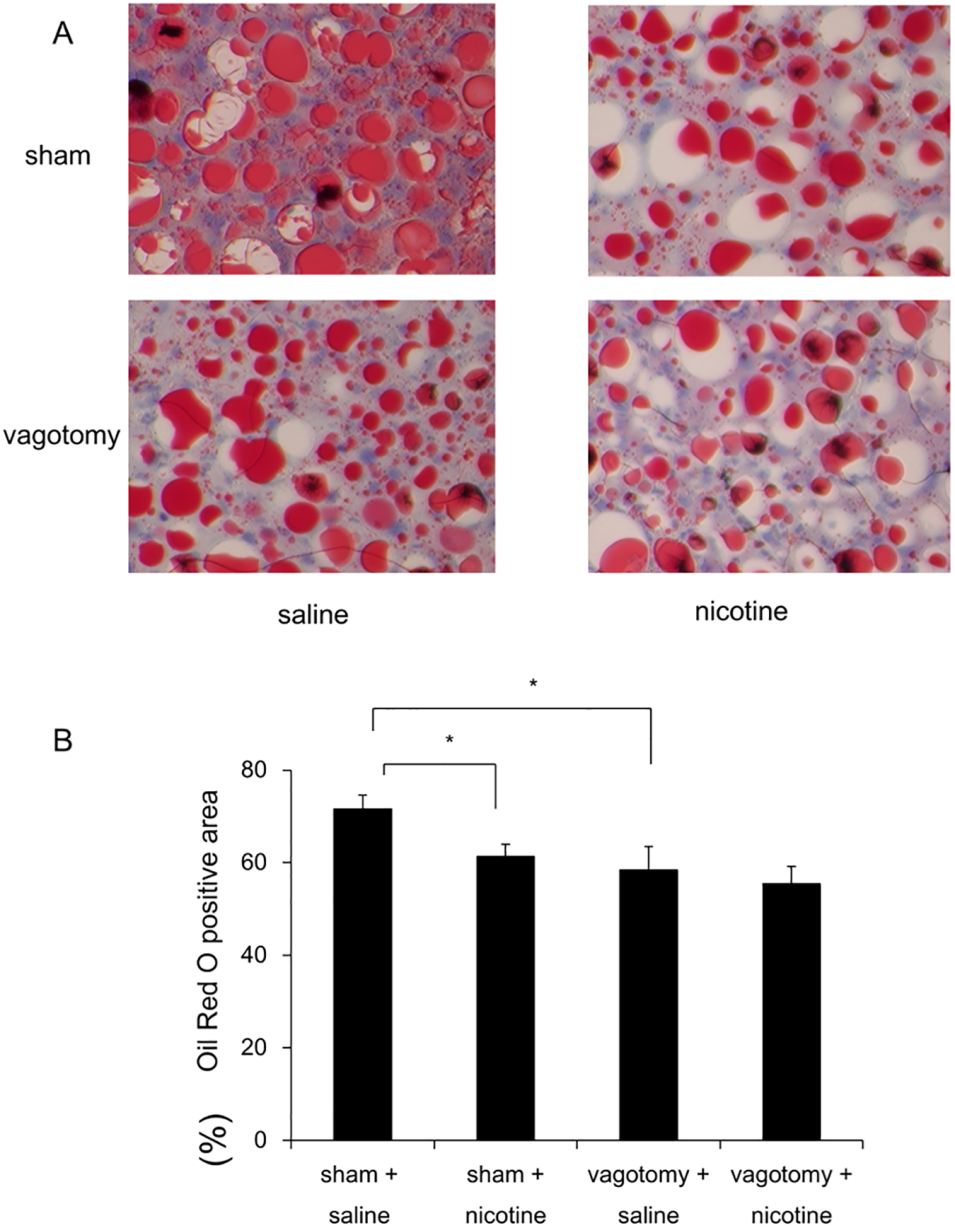
Representative photomicrographs showing the effects of hepatic branch vagotomy and sham operation on CDAA-diet-induced hepatic steatosis in rats. Frozen liver sections were stained with Oil Red O (A). Quantitative analysis of changes in Oil Red O positive lesions in the respective groups (B). Original magnification, ×100. Data are expressed as means ± SE of six rats. (^a^ p < 0.05 compared with the respective groups).

Rats that had undergone hepatic branch vagotomy showed significantly attenuated body weight gain, food intake, CDAA-diet-induced hepatic steatosis, and increased levels of hepatic TG, compared to those in sham-operated saline-treated rats (Table 4, Table 5, Fig 6A and 6B). The levels of hepatic FFA were significantly decreased in rats that had undergone hepatic branch vagotomy compared to those in sham-operated rats (Table 4). The serum levels of TG were significantly increased in rats that had undergone hepatic branch vagotomy compared to those in sham-operated saline-treated rats (Table 4). Nicotine significantly attenuated body weight gain, food intake, CDAA-diet-induced hepatic steatosis, and the levels of hepatic TG and FFA in sham-operated rats, whereas it did not change those in vagotomized rats (Table 4, Fig 6A and 6B). Nicotine could not change the serum levels of TG in sham-operated or vagotomized rats (Table 4).

**Table 4:** Effect of hepatic branch of vagotomy on CDAA diet-induced NASH model.

Group	n	Body Weight gain(g)	Food Intakes (g/day)	Serum TG (mg/dl)	Hepatic TG (mg/dl)	Hepatic FFA (mEq/l)	Serum ALT (IU/L)
sham + saline	6	79 ± 3.1	12.9 ± 0.5	25.5 ± 1.5	652 ± 110	1.2 ± 0.4	194 ± 15
sham + nicotine	6	68 ± 4.9^a^	10.1 ± 1.8^a^	23.3 ± 1.5	430 ± 15^a^	0.4 ± 0.1^a^	201 ± 9.2
vagotomy + saline	6	55 ± 3.9^b^	9.1 ± 0.5^b^	35.1 ± 3.8^a^	394 ± 79 ^a^	0.7± 0.2^a^	175 ± 16
vagotomy + nicotine	6	63 ± 6.6 ^a^	9.3 ± 1.1^a^	37.5 ± 4.3^a^	402 ± 36^a^	0.9 ± 0.1^a^	181 ± 19

Data are expressed as means ± SEM; Statistical comparison were made using one-way ANOVA

^a^; significant different from sham + saline 6 weeks (p < 0.05)

^b^; significant different from sham + saline 6 weeks (p < 0.01)

**Table 5:** NAFLD activity scores (NAS) of rats with hepatic branch of vagotomy on CDAA diet-induced NASH model.

Group	n	steatosis	inflammation	Hepatocyte ballooning
sham + saline	6	2.667 ± 0.042	1.600 ± 0.225	0.233 ± 0.033
sham + nicotine	6	2.375 ± 0.139^a^	1.200 ± 0.256 ^a^	0.100 ± 0.038^a^
vagotomy + saline	6	2.312 ± 0.051^b^	1.572 ± 0.325	0.213 ± 0.113
vagotomy + nicotine	6	2.229 ± 0.154	1.550 ± 0.253	0.240 ± 0.132

Data are expressed as means ± SEM; Statistical comparison were made using one-way ANOVA

^a^; significantly different from sham + saline (p < 0.05)

^b^; significantly different from sham + saline (p < 0.01)

**Fig 7:**
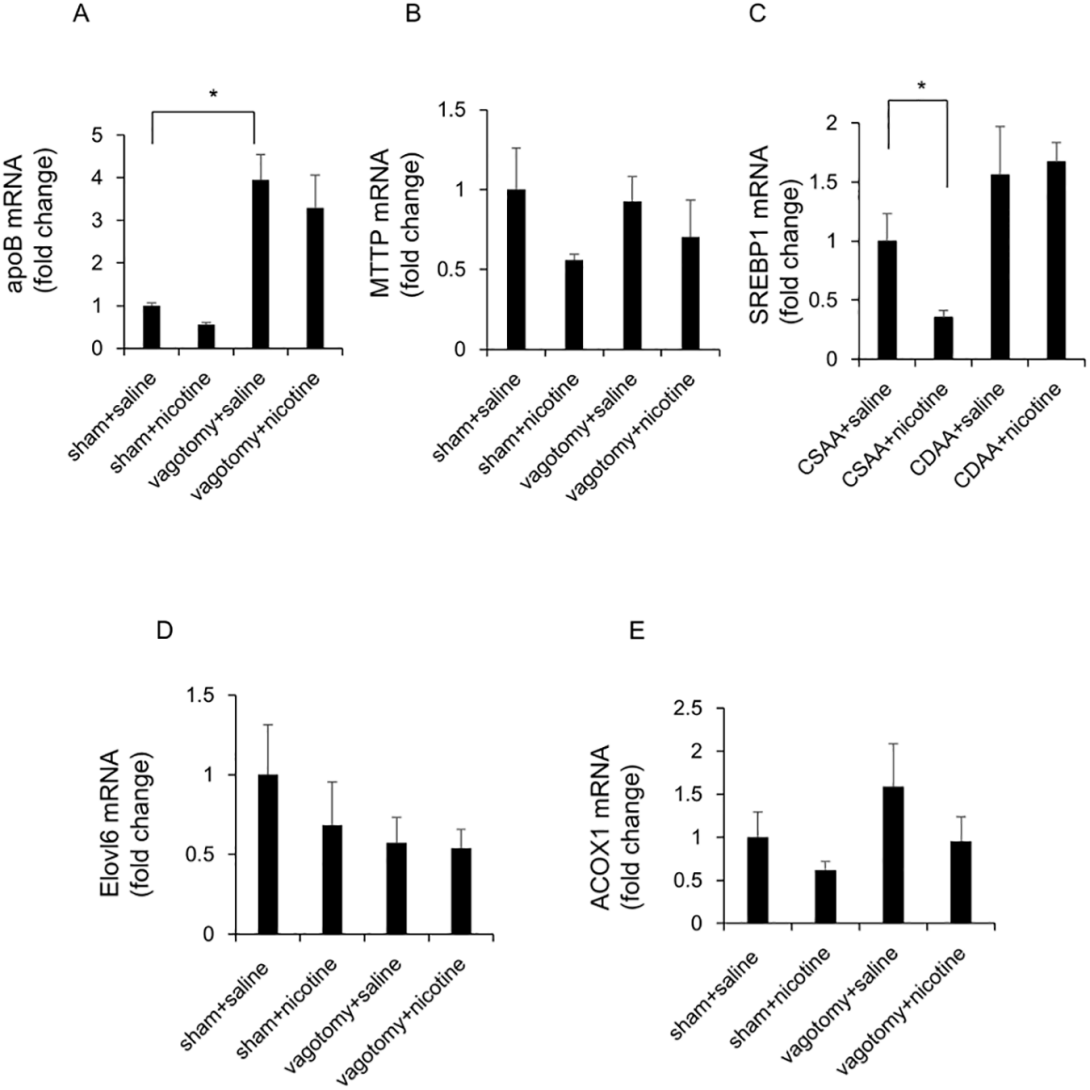
Evaluation of hepatic lipid metabolism-related gene expression in vagotomized and sham-operated rats fed with the CDAA diet. The relative mRNA expressions of *apoB* (A), *MTTP* (B), *SREBP1*(C), *Elovl6* (D) and *ACOX1* (E) were evaluated 6 weeks after the commencement of CDAA diet. The expression of target genes was normalized to that of 18s ribosomal RNA in each sample, and expressed as the magnitude of change relative to gene expression after 6 weeks of feeding CDAA diet co-administered with saline in sham-operated rats. The values are means ± SE of six rats. Statistically significant differences between the respective groups are indicated as * p < 0.05.

Hepatic branch vagotomy significantly augmented the hepatic expression of *apoB* at the mRNA level, but did not significantly change that of *MTTP*, *SREBP1*, *Elovl6*, and *ACOX1* at the mRNA level, compared to those in sham-operated saline-treated rats (Fig 7A–7E). Conversely, nicotine administration significantly attenuated the hepatic expression of *SREBP1* at the mRNA level, but could not change that of *apoB*, *MTTP*, *Elovl6*, and *ACOX1* at the mRNA level in sham-operated rats (Fig 7A–7E). Nicotine administration could not affect the hepatic expression of *apoB*, *MTTP*, *SREBP1*, *Elovl6*, and *ACOX1* at the mRNA levels in vagotomized rats (Fig 7A–7E).

**Fig 8:**
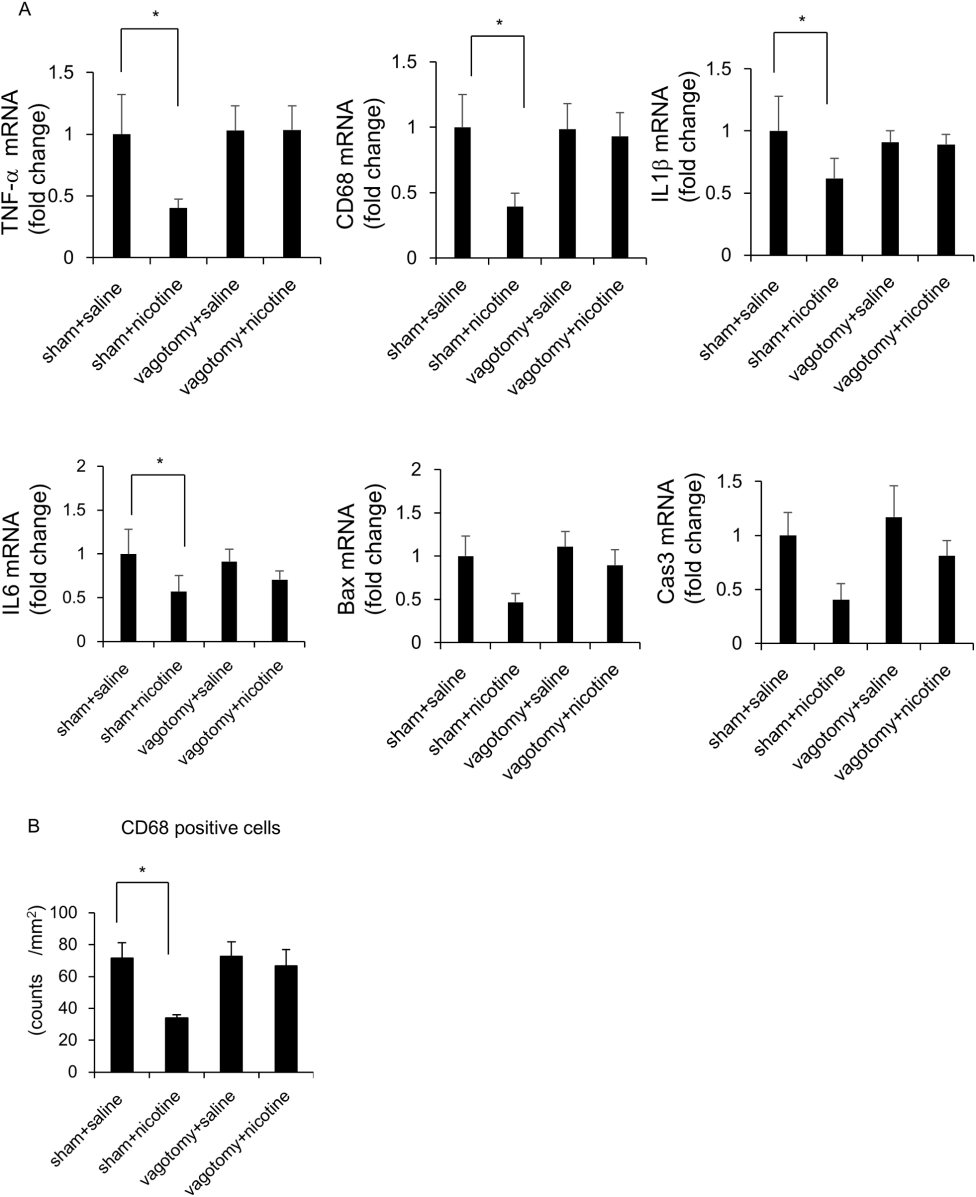
Evaluation of hepatic inflammation-related parameters in vagotomized and sham-operated rats fed with the CDAA diet. Relative mRNA expressions of hepatic *TNF-α*, *CD68*, *IL1β*, *IL6*, *Bax* and *Cas3* (A) were evaluated. The expression of target genes was normalized to that of 18s ribosomal RNA in each sample, and expressed as the magnitude of change relative to gene expression after 6 weeks of feeding CDAA diet co-administered with saline in sham-operated rats. Quantitative analysis of changes in CD68-positive cells in the respective groups (B). Data are expressed as means ± SE of six rats. (* p < 0.05, compared with the respective groups).

Hepatic branch vagotomy could not change the serum levels of ALT in saline-treated rats (Table 4), and nicotine administration could not change these levels in vagotomized or sham-operated rats (Table 4). In sham-operated rats, hepatic inflammation; ballooning scores; and expression of *TNF-α*, *CD68*, *IL1β*, and *IL6* at the mRNA levels were significantly attenuated by nicotine, whereas hepatic expression of *Bax* and *Cas3* tended to decrease (Fig 8A and 8B). Hepatic branch vagotomy could not change the hepatic inflammation; ballooning scores; expression of *TNF-α*, *CD68*, *IL1β*, *IL6*, *Bax*, and *Cas3* at the mRNA level; or the number of CD68-positive cells compared to those in sham-operated saline-treated rats (Table 5, Fig 8A and 8B). Nicotine administration could not affect the hepatic inflammation; ballooning scores; expression of *TNF-α*, *CD68*, *IL1β*, *IL6*, *Bax*, and *Cas3* at the mRNA levels; or the number of CD68-positive cells in vagotomized rats (Table 5, Fig 8A and 8B).

## Discussion

In this study, we showed that continuous administration of nicotine improved CDAA diet-induced hepatic steatosis and inflammation, and decreased the levels of hepatic TG, FFA, and the number of CD68-positive macrophages. We also demonstrated that nicotine administration significantly reduced body weight gain and food intake in CDAA diet-fed rats. A previous report indicated that nicotine administration improves obesity, hepatic steatosis, and serum lipid profile by decreasing food intake in high fat diet (HFD)-induced obese rats [11]. Conversely, Friedman et al. showed that HFD, co-administered with nicotine, produced higher levels of lipid accumulation in the liver compared to those fed with HFD alone [18]. While Friedman et al. administered nicotine at a dose of 0.75 mg/kg, which could not change food intake, other investigators administered nicotine at doses of at least 1.5 mg/kg, which attenuates food intake [11, 18, 19]. These differences in the doses of nicotine may affect food intake in rodents.

Ghoshal et al. reported that choline-deficient methionine-low diet induced hepatic steatosis and necrosis in rats [20]. The CDAA diet has been modified from the choline-deficient methionine-low diet, and long-term administration of the CDAA diet has been shown to consistently induce the development of steatohepatitis and eventual fibrosis and cirrhosis of the liver [21]. The methionine and choline-deficient (MCD) diet, which is similar to the CDAA diet, also attenuates body weight gain and leads to the development of hepatic steatosis, inflammation, and fibrosis in rodents [22]. We showed that CDAA diet itself increases hepatic TG and FFA, and decreases serum levels of TG. Emerging evidence has suggested that FFA is an important source of hepatic TG content [23]. The accumulation of fat in the liver is affected by β-oxidation, *de novo* lipogenesis, and lipid excretion; however, the precise mechanisms underlying these processes are not elucidated [22]. The expression of *ACOX1*, which is a β-oxidation-related gene, was not changed by CDAA diet as well as by nicotine administration. *SREBP1* plays a key role in hepatic *de novo* lipogenesis, and *Elovl6* is also known as a target gene of the SREBP transcription factor family [24, 25].We showed that nicotine administration attenuated the hepatic expression of *SREBP1* at the mRNA level and tended to decrease *Elovl6* expression at the mRNA level in CDAA-diet-fed rats, suggesting that nicotine administration attenuated *de novo* lipogenesis in the liver. ApoB is a protein related to the production of very low density lipoprotein (VLDL); it is an important molecule involved in TG excretion from hepatocytes [26]. MTTP, which is also a regulator of lipid excretion in hepatocytes, is involved in the production of VLDL [27]. We showed that CDAA diet alone attenuated hepatic *apoB*, but not *MTTP*, and they were not attenuated by nicotine administration. These results indicate that CDAA diet-induced increase in hepatic FFA and TG contents and decrease in serum TG content might be due to the attenuation of *apoB*, and nicotine administration is not involved in the excretion of VLDL from the liver.

The CDAA diet significantly increased hepatic inflammation, expression of *TNF-α*, *IL1β*, and *IL6* at the mRNA level, and number of macrophages in the liver, whereas nicotine administration significantly attenuated these parameters. Since pro-inflammatory and anti-inflammatory cytokines were attenuated by nicotine, anti-inflammatory immune response might be altered. The altered abundance and composition of fat in the liver could modulate the biological activity of Kupffer cells, which are resident macrophages, and augment hepatic inflammation *in vivo* [28]. The number of Kupffer cells significantly increased in the liver during the initial weeks of feeding with MCD diet [28]. Increased number of Kupffer cells enhances the sensitivity to hepatotoxin, which in turn, stimulates the secretion of pro-inflammatory cytokines and chemokines, such as TNF-α. MCD diet-fed mice exhibit significant increase in the number of Kupffer cells, which secrete several pro-inflammatory cytokines, such as TNF-α, during the initial weeks of feeding with choline-deficient diet [28]. In the present study, the CDAA diet increases the number of macrophages and levels of pro-inflammatory cytokine genes; nicotine administration may attenuate this increase resulting in the reduction of hepatic inflammation. Emerging data have shown that a high rate of hepatocyte apoptosis in patients with NASH, with the magnitude of apoptosis correlating to hepatic inflammation, indicated that apoptosis could be involved in causing NASH [29]. It has been shown that Bcl-2 family members, including both anti-apoptotic and pro-apoptotic proteins, could be major candidates of cell apoptosis [30]. We showed that the expression of *Bax*, a pro-apoptotic gene, in the liver was significantly augmented by CDAA diet, and it tended to increase *Cas3*, which is major effector caspase. Nicotine administration significantly attenuated CDAA-induced augmentation of *Bax* mRNA, and tended to decrease *Cas3* mRNA levels. Nicotine might have a potential as the inhibitor of apoptotic signaling in NASH models. Although hepatic inflammation was attenuated by nicotine, serum ALT levels were not significantly changed. NASH can occur without an increase in the liver enzyme levels, and there may not be progression of NASH in patients with fatty liver and high level of liver enzymes [31]. In the present study, high rate of fat deposition in the liver might contribute to serum ALT elevation. Considering CDAA diet-induced hepatic fibrosis, nicotine administration attenuated hepatic fibrosis, *Acta2* mRNA levels, and α-SMA positive cells. On the contrary, little is known about the effect of nicotine on stellate cell activation. Thus, it is speculated that the decrease in CDAA diet intake might contribute to the attenuation of stellate cell activation resulting in the reduction of hepatic fibrosis.

The hepatic branch of the vagus nerve plays an important role in regulating food intake [12]. Niijima et al. reported that the hepatoportal-central feedback system, acting via the hepatic branch of the vagus nerve, is an important pathway for sensing peripheral nutrients or chemicals that modulate the feeding activity [12]. Nicotine acts peripherally via the hepatic vagal afferents from nicotine sensors in the hepatoportal region [13]. In the present study, nicotine-induced inhibition of food intake was not changed in vagotomized rats, indicating that nicotine affects via the hepatic branch of the vagus nerve. The body weight reduction induced by CDAA diet is larger than that induced by vagotomy. CDAA diet-induced body weight reduction might be affected by the impairment of liver metabolism without reduction of food intake [21]. Since both vagotomy and CDAA diet alone induced body weight reduction, the level of nicotine-induced reduction of food intake required for reduction of body weight remains to be studied. We showed that hepatic branch vagotomy alone attenuated food intake during the administration of the CDAA diet, but did not change the hepatic expression of *SREBP-1*, *Elovl6*, and *ACOX1* at the mRNA level. We also showed that nicotine could not reduce food intake and change the hepatic expression of *SREBP-1*, *Elovl6*, and *ACOX1* at the mRNA level in vagotomized rats. These results indicate that the impairment of the hepatic vagal tone may affect food intake, but not *de novo* lipogenesis and β-oxidation in the liver. The hepatic expression of *apoB*, but not *MTTP*, at the mRNA level was augmented by hepatic branch vagotomy, indicating that impairment of the vagal tone may affect *apoB*-related hepatic excretion of TG from the liver.

While hepatic branch vagotomy reduced food intake, body weight gain, and hepatic steatosis, increase in CDAA diet-induced hepatic inflammation, inflammatory cytokines, and expression of apoptosis-related genes were not improved. The hepatic vagus nerve is composed of 20% efferent and 80% afferent fibers; it regulates hepatic circulation and glucose metabolism via efferent fibers, which send signals to the peripheral regions of the liver [32, 33]. The efferent vagal nerve attenuates an endotoxin-induced release of TNF-α from the Kupffer cells in the liver [34]. On the contrary, acetylcholine, which is the main neurotransmitter in the vagus nerve, reportedly suppresses the secretion of inflammatory cytokines via the α7-nicotinic acetylcholine receptor in the Kupffer cells [34]. α7-nicotinic acetylcholine receptors have been reported to be necessary in mediating activation of the cholinergic anti-inflammatory pathway by nicotine [34]. These findings helped us speculate that nicotine affects α7-nicotinic acetylcholine receptors, resulting in the attenuation of CDAA diet-induced inflammation. Hepatic vagotomy alone may impede the efferent vagal nerve-induced anti-inflammatory response.

In conclusion, nicotine attenuated food intake, body weight gain, hepatic steatosis, and inflammation in CDAA diet-fed rats via the hepatic branch of the vagus nerve. These findings indicate that nicotine may have an inhibitory effect on steatohepatitis in the rodent model of NASH.
